# O.1.1-10 Determinants of mental health: do body mass index, perceived stress and acculturation impact the mental health of international medical students?

**DOI:** 10.1093/eurpub/ckad133.086

**Published:** 2023-09-11

**Authors:** Afriza Umami, Regina Molnár, Edit Paulik

**Affiliations:** Department of Public Health, Albert Szent-Györgyi Medical School, University of Szeged, Hungary; Stikes Muhammadiyah Bojonegoro, Indonesia; umami.afriza@med.u-szeged.hu; Department of Public Health, Albert Szent-Györgyi Medical School, University of Szeged, Hungary; Department of Public Health, Albert Szent-Györgyi Medical School, University of Szeged, Hungary

## Abstract

**Background:**

International medical students are more likely to experience mental health issues. In addition to the acculturation challenges, people living with obesity often struggle with mood and anxiety disorders. The aim of this study was to investigate the association between mental health and nutritional status of international medical students, focusing on body mass index (BMI), perceived stress and acculturation.

**Methods:**

This was a cross-sectional study among international medical students at the University of Szeged, Hungary between April and October 2021. A self-administered survey was used to obtain the data. Questionnaires were asked regarding the perceived stress scale (PSS), the modified Stephenson multigroup acculturation scale (SMAS), BMI, and sociodemographic characteristics. The data were analysed using SPSS 28.0 with a multiple logistic regression model.

**Results:**

A total of 326 international medical students participated in the study. 49.7% of the students reported poor mental health and 5.2% were underweight and 10.1% were obese. We have found that perceived stress and acculturation were associated with poor mental health after adjusting for covariates. Higher perceived stress was likely to increase poor mental health (OR = 8.50; 95% CI = 3.82 – 8.93, P < 0.001), similar to acculturation where marginalization was more likely to have poor mental health (OR = 6.42; 95% CI = 2.39 – 9.03, P = 0.011). Meanwhile, the association between poor mental health and BMI in total was not significant, but underweight students were less likely to have poor mental health in the multivariable analysis (OR = 0.23; 95% CI = 0.06 – 0.86, P = 0.029).

**Conclusions:**

The results of the study can be used to advise academic institutions to help international students integrate into the local students and local communities. The arts and other free time activities, and sports could help to alleviate mental conditions including anxiety, depression, and burnout.

